# Heavy Atom Detergent/Lipid Combined X-ray Crystallography for Elucidating the Structure-Function Relationships of Membrane Proteins

**DOI:** 10.3390/membranes11110823

**Published:** 2021-10-27

**Authors:** Shinya Hanashima, Takanori Nakane, Eiichi Mizohata

**Affiliations:** 1 Department of Chemistry, Graduate School of Science, Osaka University, 1-1 Machikaneyama, Toyonaka 563-0043, Japan; hanashimas13@chem.sci.osaka-u.ac.jp; 2 Department of Biological Sciences, Graduate School of Science, The University of Tokyo, Tokyo 113-0033, Japan; tnakane@mrc-lmb.cam.ac.uk; 3 Department of Applied Chemistry, Graduate School of Engineering, Osaka University, 2-1 Yamadaoka, Suita 565-0871, Japan; 4 Japan Science and Technology Agency, PRESTO, Kawaguchi 332-0012, Japan

**Keywords:** membrane protein, lipid, detergent, chemical synthesis, X-ray free-electron laser (XFEL), serial femtosecond crystallography (SFX), structure-function relationship

## Abstract

Membrane proteins reside in the lipid bilayer of biomembranes and the structure and function of these proteins are closely related to their interactions with lipid molecules. Structural analyses of interactions between membrane proteins and lipids or detergents that constitute biological or artificial model membranes are important for understanding the functions and physicochemical properties of membrane proteins and biomembranes. Determination of membrane protein structures is much more difficult when compared with that of soluble proteins, but the development of various new technologies has accelerated the elucidation of the structure-function relationship of membrane proteins. This review summarizes the development of heavy atom derivative detergents and lipids that can be used for structural analysis of membrane proteins and their interactions with detergents/lipids, including their application with X-ray free-electron laser crystallography.

## 1. Introduction

All cellular organisms have biomembranes composed of lipids, which form a boundary between the cell cytoplasm and the surrounding extracellular environment and organize complex structures inside the cell to construct cellular organelles [1]. Membrane proteins localize in the lipid bilayer of biomembranes and play a variety of important functions such as transport of various substances between the inside and outside of the cell, signal transduction, energy synthesis, and cell adhesion. In particular, membrane proteins associated with human diseases are attracting considerable attention as drug targets and are actively studied. Compounds that bind to membrane proteins and inhibit or promote their functions are being explored as drug candidates, and membrane proteins overexpressed on the surface of certain cancer cells are targets for the development of antibody drugs [2,3,4,5]. Thus, elucidation of the structure and function of membrane proteins including their interactions with lipids at the molecular level is essential both from the perspective of basic science to understand the mechanisms of life and from the perspective of medical applications. However, the progress of membrane protein research has lagged behind that of water-soluble proteins because of the difficulties associated with isolating hydrophobic membrane proteins in sufficient quantity and quality for analysis. In the field of structural biology, only about 6000 of the approximately 180,000 structural coordinates registered in the Protein Data Bank (PDB) are those of membrane proteins.

Nonetheless, advances in research technology have accelerated the study of membrane protein structures. With the continuous development of genetic engineering, expressing high levels of stable membrane proteins as recombinant proteins and purifying them with a sufficient yield has gradually become possible. High-speed atomic force microscopy has made it possible to image motion during functioning with nanometer-order spatial resolution without destroying the structure of biological samples [6]. Cryo-electron microscopy has undergone remarkable technological improvements over the last decade, enabling structural analysis of membrane proteins and their complexes with near-atomic resolution without requiring crystal preparation [7]. Most attempts to visualize the three-dimensional structure of membrane proteins and their interactions with lipids/detergents at near-atomic resolution or better have been made using X-ray crystallography. Advancing synchrotron radiation facilities and beamline technologies have facilitated the determination of crystal structures of membrane proteins. This trend will continue with the development of fourth-generation synchrotron radiation sources, including X-ray free-electron laser (XFEL) facilities [8].

Solving new structures by X-ray crystallography requires both intensities and phases of structure factors of diffracted X-ray waves; however, measured diffraction patterns only give intensities (so-called “phase problem”). Therefore, experimental (de novo) phase determination methods including single or multiple heavy-atom isomorphous replacement (SIR or MIR), single- or multi-wavelength anomalous diffraction (SAD or MAD) or the combination of SIR or MIR with anomalous scattering (SIRAS or MIRAS) must be applied to solve the phase problem. Isomorphous replacement methods use the difference in reflection intensities between the native crystal and the heavy atom-labeled derivative crystal to determine the phases, whereas the anomalous diffraction method uses anomalous differences between Bijvoet pairs [9]. Both methods require accurate measurement of small intensity differences. However, expressing selenomethionine-labeled recombinant proteins is time-consuming and costly, and even if the native crystals are soaked in a heavy-atom solution, the heavy atoms often do not bind to the protein or the crystallinity easily collapses, resulting in loss of diffraction ability. Thus, the development of heavy atom labeled protein ligands is needed that bind various membrane proteins and facilitate drawing electron density maps efficiently from X-ray diffraction data to reveal three-dimensional structures.

In this review, we summarize the development and use of heavy atom derivative detergents/lipids that can be used to analyze interactions between membrane proteins and detergents/lipids and to determine the structure of membrane proteins. We also discuss the potential of combining recently developed XFEL crystallography with heavy atom detergents/lipids to understand the structure-function relationship of membrane proteins and their interactions with detergents/lipids.

## 2. Heavy Atom Labeled Protein Ligands

Typical de novo methods to determine crystallographic phases involve heavy-atom derivatization of protein crystals. These crystals can be used for isomorphous replacement methods and anomalous diffraction methods. The period 4 to 6 elements, including lanthanides, are often used as electron-rich heavy atoms for solving phase problems when determining protein structures by X-ray crystallography. Soaking manipulation introduces heavy metal ions or heavy atom-labeled compounds into protein crystals. The successfully soaked ions or compounds in protein crystals are immobilized through interactions with functional groups of proteins mostly by ionic and dipole linkages, H-bonding, and coordination bonding [10,11]. Numerous conditions are typically screened as a rule of thumb to ensure successful crystallization of the target protein and referring to the chemistry of ionic interactions and covalent modifications is often useful for the introduction of heavy atoms into protein crystals.

Protein ligands containing heavy atom moieties have been developed to achieve two purposes. First, these ligands unveil the molecular interactions between proteins and ligands. The strong and characteristic diffraction of heavy atoms, due to their high electron density and anomalous scattering, reveals the ligand position on the protein. Second, the ligand can be used for de novo phasing to determine the protein structure. However, careful design of the heavy atom ligand is essentially required to avoid disturbance for the protein-ligand interaction. Heavy atom-containing detergents/lipids are potentially useful for solving structures of membrane proteins because they have affinity for the hydrophobic surface of membrane proteins and can be used to determine phases.

### 2.1. Halogens

Protein-ligand analogs carrying a Br or I atom have been used successfully to determine X-ray protein crystal structures with de novo phasing; however, careful design of the analog structure is important because of the properties of halogen atoms. The carbon(*sp*^3^)–halogen bond length (C–Br: 191 pm; C–I: 216 pm) is significantly longer than the corresponding carbon–carbon bond length (151 pm) [12]. Thus, careful consideration may be required when selecting protein crystallization conditions because of the bulkiness and lower bond-dissociation energy of the carbon(*sp*^3^)–halogen bond (C–Br: 72.4 kcal/mol; C–I: 56.9 kcal/mol) [13]. Possible instability can be overcome by installing halogens on an aromatic ring because the bond-dissociation energy between a benzene carbon (Ph) and the halogen atom is higher (Ph–Br: 84 kcal/mol; Ph–I: 67 kcal/mol) than that of carbon(*sp*^3^)–halogen bonds [13].

The high electron density and anomalous scattering from halogen atoms are used to disclose the exact atom position in crystal structures. A powerful application is to elucidate the binding of drugs to membrane proteins. For example, the anomalous scattering of Br installed on Fluoxetine (Br–Fluoxetine), the antidepressant drug named Prozac^®®^, clearly reported the ligand position at the fenestration of the K2P channel TREC-2, which is regulated by arachidonic acid [14]. In a similar approach, Br-labeled Memantine, a drug used for treating Alzheimer’s disease, was used to accurately unveil the binding mode to the prokaryotic pentameric ligand-gated ion channel [15]. The crystal structure indicated that the Br analog blocks the extracellular entryway of the channel pore to inhibit ion passage through the channel.

Halogen labeling has also been used to determine the crystal structure of RNA molecules. RNA is a biopolymer that plays a direct role in the regulation of cellular activities through molecular interactions. Some structures of RNA have been successfully determined through de novo phasing using halogen atoms such as 5-bromouridine [16] and 5-iodouridine [17]. Nucleotides are crucial protein substrates for maintaining cellular homeostasis. ATP mimicking analogs with a halogen atom are good inhibitors of protein kinases. The affinity of protein-inhibitor binding is dependent on the number and position of halogen substitutions, which contribute to “halogen bonding” [18]. Therefore, halogen bonding is another important function of halogen atoms introduced on the protein ligand, which may enhance ligand-protein interactions and possibly modulate the orientation of the ligand [19].

**Figure 1:**
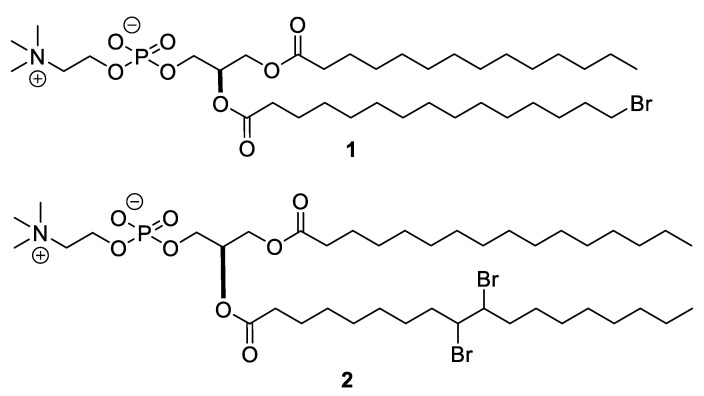
Structures of the bromo-phospholipids.

Cell membrane lipids are indispensable for regulating the structure and function of membrane-related proteins. Phospholipids carrying brominated hydrocarbon chains **1** and **2** have been used to examine phase separation and domain distributions (Figure 1) [20,21,22]. Namely, an electron-rich Br atom selectively positioned in the middle or terminal position of a phospholipid hydrocarbon chain efficiently acts as a collisional quencher for a fluorescence group close to the Br atom. These brominated fatty acids of phospholipids are readily prepared by substituting the terminal alcohol with Br [23] or adding Br_2_ to an unsaturated bond [24]. The electron-rich Br atom can be a good reporter in X-ray diffraction studies for detecting particular atomic positions in lipid tails consisting of continuous methylene and terminal methyl groups. X-ray diffraction data of a bilayer membrane composed of mono-bromo dipalmitoylphosphatidylcholine (DPPC) **1** disclosed the terminal position of the hydrocarbon chain in the membrane, where the terminus of lipid chains was substituted with a Br instead of the ω-methyl group [25]. Br mimics the size of the ω-methyl group of the hydrocarbon chain because both have similar van der Waals radii (Br: 185 pm; CH_3_: 200 pm). Bromination did not affect the transition enthalpy and cooperativity significantly; although, mono-bromo DPPC showed a significant decrease by 9 °C in the phase transition temperature. Brominated lipids were also used to determine lipid binding sites on the surface of membrane proteins in single-crystal X-ray structural studies of proteins [26]. This was achieved by discriminating between the hydrocarbon chains of lipids and those of detergents that were used to reconstitute membrane proteins into membranes. When labeling phospholipids with Br atoms, the anomalous signals from dibromo phospholipid **2** in Figure 1 and its analogs with different lipid headgroups make it easier to discriminate lipid electron densities of surrounding lipids from those of detergents and other molecules.

**Figure 2:**
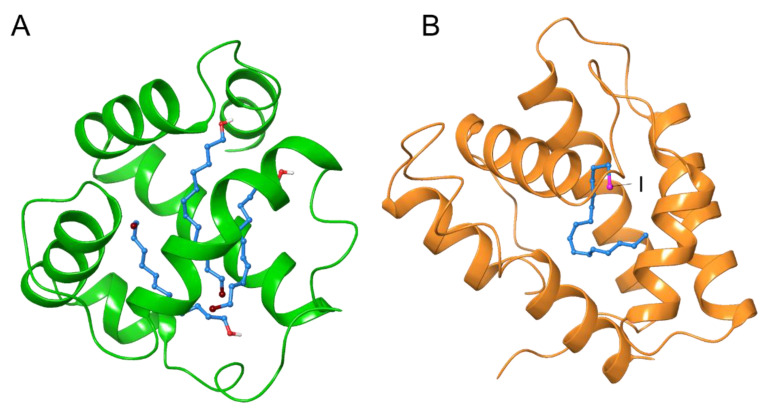
Crystal structures of the complex with lipidic ligands with halogen moiety. Moth chemosensory protein CSPMbraA6 with bromohydrocarbon chain (PDB ID: 1N8U) (**A**). Moth pheromone binding protein BmorPBP with iodohexadecane (PDB ID: 2P71) (**B**). Hydrocarbon chain of the ligands were shown in sky-blue, and Br and I were shown in purple and magenta, respectively.

Brominated lipids can be used to determine lipidic ligand–protein interactions (Figure 2). Chemosensory proteins transport chemicals from air to the receptor and are associated with olfaction or taste processes. 12-Bromododecanol was used in a crystallization study as a surrogate for the hydrophobic ligand of a chemosensory protein [27]. The clear observation of three peaks in the anomalous difference electron density map revealed that three chain positions of the bromohydrocarbon occupied the large ligand cavity of the protein after major structural changes. Pheromone binding proteins are another class of odorant proteins. Similarly, iodohexadecane was used instead of the intrinsic pheromone, bombykol, to determine the conformation of the binding cavity of the insect pheromone binding protein from *Bombyx mori* [28].

**Scheme 1:**
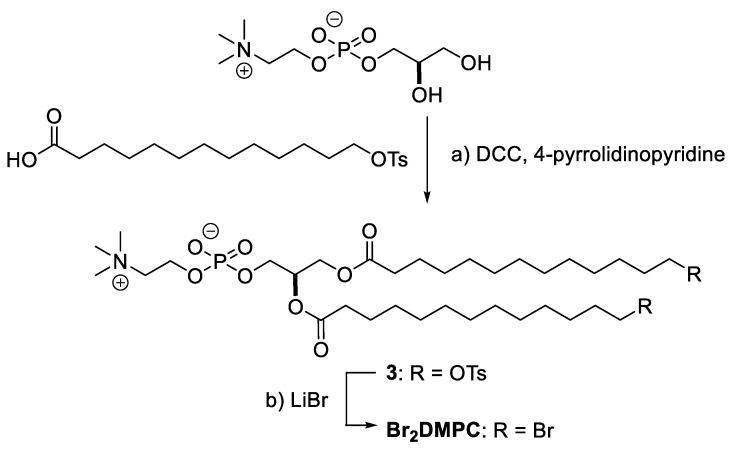
Synthesis of ω-Br_2_-DMPC. Synthetic procedures; (**a**) *N*,*N*’-dicyclohexylcarbodiimide (DCC), 4-pyrrolidinopyridine, CH_2_Cl_2_, 40%. (**b**) LiBr, acetone 41%. Ts; *p*-toluenesulfonyl group. Detail of this synthesis was described in Supplementary Materials.

We recently developed the synthetic route for ω-doubly brominated 1,2-dimyristoyl-*sn*-glycero-3-phosphocholine (Br_2_-DMPC) via the tosyl intermediate (Scheme 1). Esterification of the ω-tosyl fatty acid to the two hydroxy groups on glycerophosphocholine, instead of using the ω-bromo fatty acid, successfully produced an intermediate **3** in moderate yield, and the following substitution reaction using LiBr afforded Br_2_-DMPC. These brominated lipids will be used as ligands and as annular lipids in membrane protein crystallization studies.

**Figure 3:**
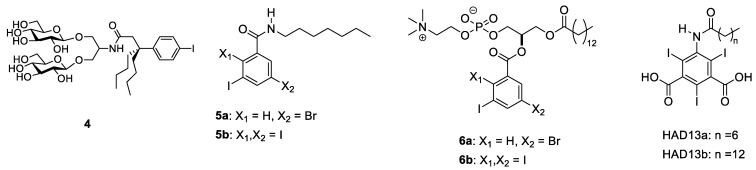
Lipophilic compounds and detergents bearing multiple halogen substitutions that can be used to solve the phasing problem for membrane protein structures.

Large membrane proteins usually consist of multiple transmembrane helices and sizable ectodomains. Therefore, labeling multiple sites of the membrane protein with heavy atoms is an approach to solve phase problems in X-ray diffraction studies. The use of appropriate detergents is often required to manipulate and crystallize membrane proteins. Detergents used for crystallization can be conveniently labeled with heavy atoms, which is suitable for de novo phasing. Accordingly, heavy atom-bearing tripod amphiphiles shown in Figure 3 were developed previously [29]; the iododetergent **4**, which solubilizes membrane proteins, is suitable for manipulating membrane proteins.

We developed amphipathic compounds, phospholipid mimics and detergents bearing multiple halogen substitutions on aromatic rings for de novo phasing and revealing lipid-protein interactions. The amphipathic properties manifested by the hydrophilic headgroups linked to the lipophilic hydrocarbon tails are essential for these molecules to mix well with bilayer membranes and interact with membrane proteins in a similar manner to intrinsic lipids (Figure 3). Furthermore, the multiple halogens arrayed on a plane of the aromatic ring assisted with the easy detection of the moiety. Commercially available benzoic acid or phthalic acid derivatives carrying multiple halogen substitutions, some of which have been used as a phasing reagent in protein crystallization [30,31], were coupled with lipophilic moieties through amidation or esterification with alkyl amine, lysophosphatidylcholine or fatty acids to give compounds **5a**, **5b**, **6a** (HAD16), **6b**, HAD13a [32], and HAD13b (see Supplementary Materials for synthetic details). Importantly, HAD13 was developed based on a successful phasing reagent 5-amino-2,4,6-triiodoisophthalic acid (I3C, or magic triangle) [30,31] for application in X-ray imaging. I3C is a common synthetic intermediate for several X-ray contrasting reagents safely used to improve the visibility of vascular structures and organs during radiographic procedures in clinical diagnostics [33,34]. I3C has been used for solving the crystal structure of more than 30 protein structures (mainly soluble proteins) in the PDB. Recently, 5-amino-2,4,6-tribromobenzene-1,3-dicarboxylic acid and tetrabromoterephthalic acid, which are bromo analogs of I3C, were successfully used as phasing reagents in the crystallization of model proteins [35,36]. Further application of I3C and other halogenated benzoates for membrane protein crystallization was achieved by improving the amphiphilicity of the halogenated benzoate by introducing an acyl chain at an amino group or coupling with a lysophospholipid. The amphiphilic nature of these compounds ensures good miscibility with phospholipids and for acting as detergents by surrounding membrane proteins. Phospholipid mimics **6a** (HAD16) and **6b** and detergents HAD13a and HAD13b were miscible in DMPC/CHAPSO bicelles (*q* = 2.8), which were used for crystallization of membrane proteins [37], whereas hydrophobic compounds **5a** and **5b** were shown to readily form precipitates. In practice, HAD13a was used successfully to perform de novo phasing of XFEL diffraction data obtained from membrane protein microcrystals (see details in Section 3).

### 2.2. Selenium

Selenium is a major heavy atom that is used to achieve de novo phasing of protein crystallographic data. Oxygen and sulfur belong to the second and third periods of the group 16 elements in the periodic table, thus being potentially substituted with a selenium element in the 4th period. Therefore, recombinant expression using selenocysteine and selenomethionine readily furnishes target proteins with heavy atom labels, which are suitable for the SAD/MAD phasing method [38]. However, care must be taken because the toxicity of selenomethionine sometimes hampers the growth of recombinant organisms used to overexpress target proteins. In addition, selenium derivatives of nucleic acids have been incorporated into DNA and RNA to solve the phase problem in MAD phasing [39].

Selenium-labeled derivatives of ligands that bind to proteins have been synthesized for protein crystallography. The selenium atom on the protein ligand is used to phase X-ray diffraction data and determine the exact atomic position of the bound ligand by using anomalous scattering, as is the case with halogen ligands. In carbohydrate chemistry, selenium-containing sugars and their derivatives have been developed as antioxidants and glycosidase inhibitors [40,41]. Although selenium is a highly toxic element, the potential of these seleno-sugars as lead compounds for therapeutic reagents has been examined [42].

**Scheme 2:**
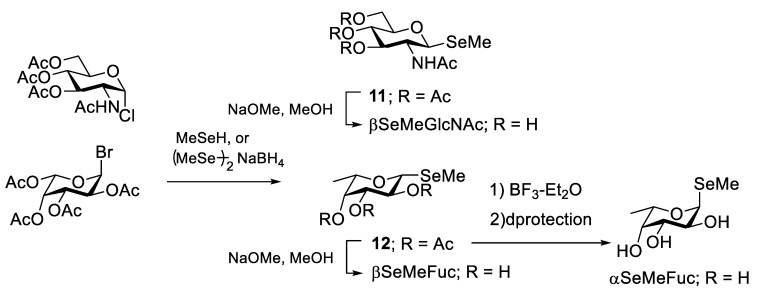
Synthetic scheme of the selenoglycosides where selenium was substituted at the glycoside oxygen.

**Figure 4:**
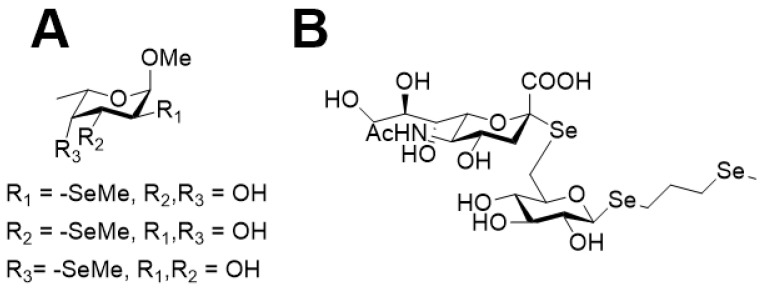
Fucose derivatives with selenomethyl substitutions at the 2, 3, or 4 hydroxy group (**A**) and sialylglucose derivative with three selenomethyl substitutions (**B**).

Selenomethyl-*N*-acetylglucosamine (*β*MeSe-GlcNAc), a selenium-labeled sugar, was first used for protein crystallization to solve the phasing of data obtained on bacterial F17-G adhesin (Scheme 2) [43] because GlcNAc is a native ligand of this adhesin. The selenomethyl group was smoothly installed at the anomeric position of 1-chloro-GlcNAc. *β*MeSe-GlcNAc successfully mimics the native ligand because the binding constants of GlcNAc and *β*MeSe-GlcNAc to the adhesin are similar. Imberty and co-workers developed a selenomethyl derivative of fucose (*β*-MeSe-Fuc) (Scheme 2). *β*MeSe-Fuc was first used as a seleno-ligand for phasing when solving the crystal structures of fucose-binding lectins derived from a plant pathogenic bacteria [44]. *β*MeSe-Fuc was also used to determine the co-crystal structure of other lectins AFL [45] and BC2L-C [46], which originate from the opportunistic infection-related fungus *Aspergillus fumigatus* and the bacterium *Burkholderia cenocepacia*, respectively. αMeSe-Fuc was readily synthesized by anomerization of βMeSe-Fuc with a Lewis acid [47] or glycosylation between fucosyl imidate and a selenoacetal acceptor [48]. Recently, Shimabukuro et al. successfully introduced the MeSe group at the 2–, 3–, or 4–OH position of fucose (Figure 4) and these compounds were used as ligands for a fucose binding lectin that originated from *Aspergillus oryzae* [49]. The MeSe substitution disclosed the essential hydroxy group for interaction with the lectin. Currently, the selenium atom can be incorporated into an oligosaccharide structure, which facilitates applications of seleno probes to elucidate oligosaccharide binding to proteins by not only crystallography but NMR because ^77^Se (I = 1/2, 7.6% natural abundance) is an NMR active nucleus that gives rise to a sharp signal [50].

**Figure 5:**
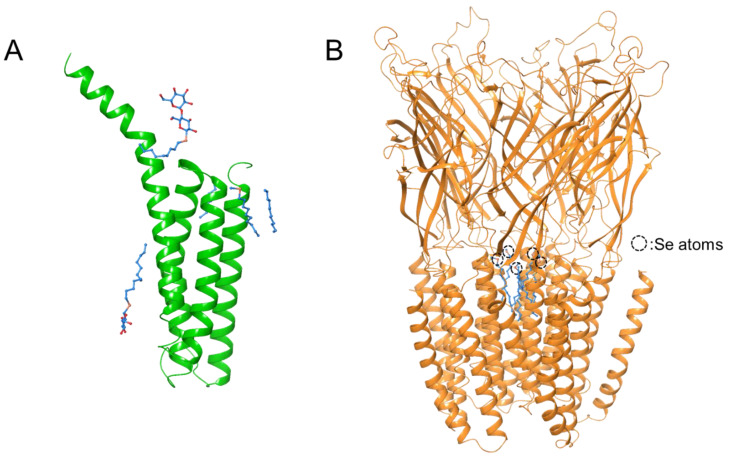
Crystal structures of transmembrane proteins Leukotriene C4 synthase (PDB ID: 3B29) (**A**) and pLGIC pentamer (PDB ID: 4IL4) (**B**) using SeDDM as the phasing detergent. The hydrocarbon chains of Se-DDM are shown in sky-blue.

The hydrophilic sugar moieties are also used as headgroups of detergents to manipulate and crystallize membrane proteins. Detergents carrying a heavy atom can be used in membrane protein studies. Dodecyl-*β*-D-selenomaltoside (SeDDM), a heavy atom analog of dodecyl-*β*-D-maltoside (DDM), was used to achieve MAD phasing when solving the structure of leukotriene C4 synthase by X-ray crystallography (Figure 5A) [51]. Furthermore, SeDDM was used successfully as a phasing detergent to solve the structure of the prokaryotic pentameric ligand-gated ion channel [52]; however, the sugar headgroups were poorly resolved (Figure 5B). Detergent bundles of SeDDM were clearly observed inside the pore.

**Scheme 3:**
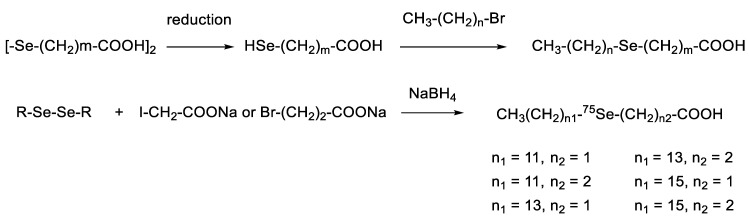
Conventional synthesis of seleno-fatty acids.

Fatty acids are amphiphilic compounds ubiquitously present in our body. A seleno-fatty acid whose methylene group was substituted with a selenium atom was developed because their van der Waals radii are very similar (Se: 190 pm; CH_2_: 200 pm). Carbon belongs to group 14 and is thus different from selenium in the periodic table. This difference results in a slightly longer bond length (C–Se: 194.5 pm; C–C: 154.0 pm) and a smaller bond angle (C–Se–C: 96.3°; C–C–C 112.6°). Thus, the distance between the two carbon atoms of a C–Se–C moiety is 289.8 pm, which is 34 pm longer than the distance of 1,3-carbon atoms in an alkane. However, this difference does not affect the conformation of the whole acyl chain significantly, except for the selenoether and its neighboring portions. Therefore, selenium can be used as a bioisostere for a methylene unit, which is particularly useful for introducing a heavy atom into the hydrophobic part of detergents and ligands. For example, Fredga and Lindgren first reported the synthesis of 4-seleno-hexadecanoic acid and 12-seleno-hexadecanoic acid, the seleno analogs of palmitic acid (Scheme 3) [53]. Two or more selenium atoms were also incorporated into a fatty acid using a similar synthetic pathway [54]. Sadek and Basmdjian incorporated radioactive ^75^Se into fatty acid chains for imaging [55].

Selenium is an essential trace element and a source of the antioxidant selenoproteins. Ether-type lipids bearing a selenomethyl moiety at the terminus of a hydrocarbon chain were synthesized to gain antioxidant activity [56,57]. Interestingly, seleno fatty acids were also developed as antimicrobial agents [58]. These seleno-lipid derivatives can be used to solve the phase problem in appropriate protein crystallization studies.

**Figure 6:**
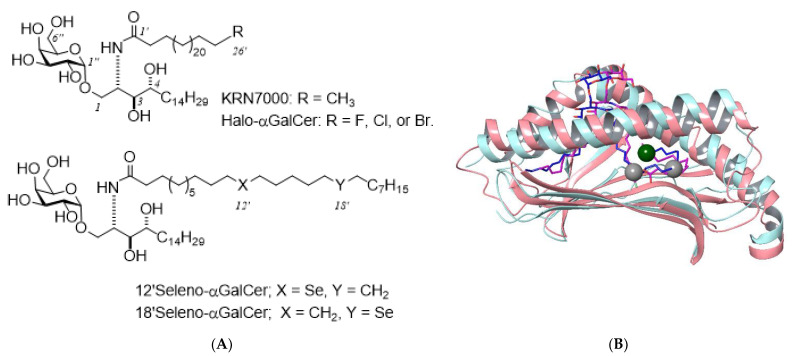
Structures of heavy atom derivatives of α-galactosylceramide and binding model with CD1d. The chemical structures of α-GalCer derivatives with terminal halogens or selenium (**A**), and the superposed structures of murine CD1d complexed with Th1-selective ligand C8PhF (PDB ID: 3GMO; pink ribbon and purple wire) and that with KRN7000 (PDB ID: 3HE6; sky-blue ribbon and blue wire) (**B**). C26′ of KRN7000 is shown as a dark green ball and C12′/C18′ are shown with gray balls, which corresponded to halogen and selenium positions of the synthetic analogues, respectively.

Lipidic ligands carrying a heavy atom moiety are also useful for revealing lipid–protein interactions, not only for phase determination but by making the heavy atom position conspicuous with the anomalous difference map. Recently, we developed a heavy atom derivative of α-galactosylceramide (α-GalCer) known as KRN7000, which activates immune responses by inducing cytokine production upon binding to the protein CD1d (Figure 6) [59]. Selenium was incorporated into the fatty acid chain of α-GalCer by substitution of a methylene group through chemical synthesis. The selenium and ω-halo derivatives were potent inducers of IFN-γ and IL-4 production in murine splenocytes. The heavy atom-modified lipid derivatives of α-GalCer were accommodated deeply in the lipid-binding cavity of CD1d.

## 3. Serial Femtosecond Crystallography (SFX)

An important recent innovation in quantum beam technology for structural biology is the emergence of XFEL. The world’s first XFEL facility, LCLS [60], was built at Stanford University in the United States in 2009. In 2011, SACLA [61] was established in Japan, followed by the European XFEL [62] in Germany, PAL-XFEL [63] in Korea and SwissFEL [64] in Switzerland. Serial femtosecond crystallography (SFX) is a new data measurement technique of X-ray crystallography, which takes advantage of the ultra-high beam brilliance, femtosecond pulse duration and high spatial coherence of XFEL [65]. At SACLA, an operation mode with a wavelength range of 0.62–2.76 Å, a pulse duration of 2–10 fs, a pulse energy ~400 uJ at 10 keV, and a frequency of 30–60 Hz is available for SFX [66].

In SFX measurements, many microcrystals are ejected at random orientations from an injector and introduced into the orbit of the XFEL beam, and the diffraction image produced when a single pulse of XFEL hits a microcrystal within a femtosecond exposure time is recorded at room temperature. Because the brightness of the XFEL is typically a billion times brighter than synchrotron radiation such as SPring-8, the diffraction intensity obtained with a single XFEL pulse is comparable to the diffraction intensity of a 1-s exposure with synchrotron radiation. Diffraction images of thousands to a half-million microcrystals are collected for structural analysis. In the case of synchrotron radiation crystallography (SRX), at least milliseconds of exposure time are required for data collection, which results in radiation damage and photoreduction, a phenomenon where hydrated electrons that are generated on the order of picoseconds by the interaction of X-rays with water molecules in the crystal react with protein molecules in the crystal to break chemical bonds and reduce metal active centers [67]. Because femtosecond XFEL diffraction in SFX is completed on a shorter timescale than the process of radiation damage and photoreduction, this technique captures the damage-free structure of samples [68].

### 3.1. De Novo Phasing in SFX

Since the first construction of the XFEL facility in 2009, all SFX structures reported in international journals had been solved by the molecular replacement method using known structures as search models. This is because phase determination by the anomalous scattering method requires accurate measurement of small intensity differences between the reflections of the Bijvoet pairs, which is much more difficult with SFX than with the conventional SRX oscillation method. In SFX, data are collected by irradiating a large number of microcrystals of different sizes and orientations with XFEL pulses that have fluctuations in intensities and wavelength spectra. This leads to large errors in the observed diffraction intensities, and all measurements are partial reflections, making it difficult to determine the phases. In 2014, the first de novo structure determined with SFX was reported by the Gd-SAD method using a model protein lysozyme [69]. Subsequently, we reported de novo phasing of the luciferin-regenerating enzyme by the Hg-SIRAS method [70] and lysozyme by the S/Cl-SAD method [71] in 2015 and successfully determined the structure of copper-containing nitrite reductase by the Cu-SAD method in 2016 [67]. In the same year, the phase determination of BinAB by the Hg/Gd/I-MIRAS method [72] and streptavidin by the Se-SAD method [73] were reported. Thus, successful cases of de novo phasing by SFX were reported. However, these data analyses are all from water-soluble proteins and required tens or hundreds of thousands of high-resolution diffraction images of 1.7–2.3-Å resolution. Therefore, the authors took on the challenge of developing an efficient method for the phase determination of membrane proteins by SFX.

### 3.2. De Novo Phasing with the HAD13a Detergent

**Figure 7:**
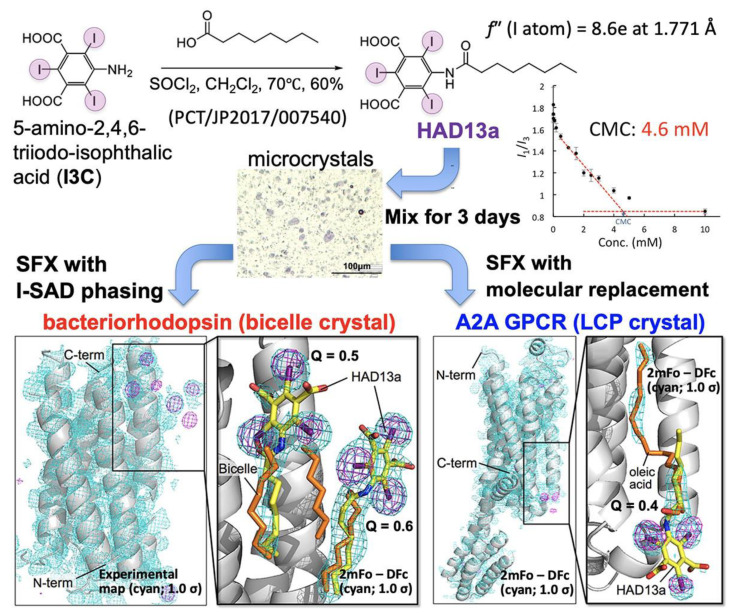
Serial femtosecond crystallography (SFX) of membrane proteins that have interacted with iododetergent, HAD13a. Figure reproduced with modification from Ref. [32].

In general, the diffraction quality of membrane protein crystals is lower when compared with that of water-soluble proteins. This is also true for SFX using the most brilliant XFEL light source available today. In the experimental phasing method of SRX, heavy atoms such as Se, Hg, Au, and Pt, which have absorption edges around 1.000 Å, have been selected frequently and used to derivatize relatively large crystals (50–200 μm). This is because, in most cases, the beamlines used for X-ray crystallography of biological macromolecules are optimized for wavelengths around this range. In contrast, because SFX uses microcrystals (1–50 μm), X-ray beams with longer wavelengths are often selected to obtain larger diffraction signals. We selected iodine, which has an excellent anomalous scattering effect at longer wavelengths (*f*” = 8.6e at 1.771 Å). HAD13a was synthesized by attaching a hydrophobic alkyl chain (caprylic acid) to I3C to give HAD13a affinity toward hydrophobic surfaces of membrane proteins [32]. HAD13a has detergent properties (CMC: 4.6 mM) and was used to label membrane proteins with heavy atoms by simply mixing it with microcrystals of bacteriorhodopsin crystallized by the bicelle method or G protein-coupled A2a adenosine receptor (A2A GPCR) obtained by the LCP method (Figure 7). The HAD13a-labeled bacteriorhodopsin was successfully phase-determined by SAD, SIR, and SIRAS methods using iodine atoms. For phase determination by the SAD method, 23,000 indexed diffraction images and a resolution of 2.1 Å were required. In contrast, in the SIRAS method with the addition of native crystal data, reflections up to a resolution of 3.3 Å were sufficient for phasing, and when the resolution was extended, only 7000 (4000 derivative and 3000 native) indexed images were required to determine the phase. This indicates that the SIRAS method is more powerful than the SAD method for efficient de novo phasing in SFX. At a similar time as our study, Batyuk et al. succeeded in determining A2A GPCR by the S-SAD method, which required 500,000 images and obtained a resolution of 2.5 Å [74].

### 3.3. Binding of the HAD16 Lipid to a Membrane Protein

**Scheme 4:**
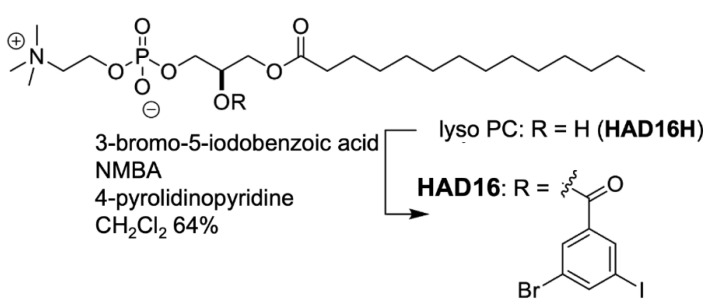
Synthetic reaction scheme for HAD16. HAD16 was readily produced via a single step synthesis by the esterification. The easy preparation will expand its application range. Details of this synthesis were described in Supplementary Materials.

HAD16 was synthesized by modifying the aromatic head group of HAD16H with multiple heavy-atom halogen groups to furnish a hydrophobic alkyl tail (Scheme 4, Supplementary Materials Section S6). The structural properties of HAD16 mimic a phosphatidylcholine structure. HAD16 can be used as a tool for analyzing lipid-membrane protein interactions. Microcrystals of bacteriorhodopsin obtained by the bicelle method were mixed with HAD16 and then SFX data were collected at SACLA (Supplementary Materials Section S7). We tried de novo phasing the HAD16 dataset. Although two heavy atom sites were located by the SIR, SIRAS or SAD method, auto-tracing was not possible even with all 18,069 lattices obtained. This is most likely because of the smaller isomorphous and anomalous signal from HAD16, which contains only one I atom and one Br atom per molecule instead of three I atoms in HAD13a, as well as only one HAD16 molecule in the asymmetric unit when compared with two HAD13a molecules.

**Figure 8:**
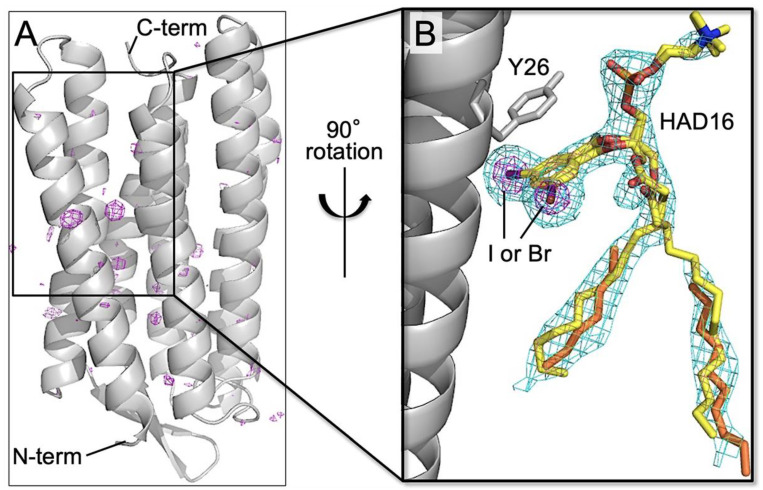
Structure of bacteriorhodopsin in complex with HAD16. (**A**) Final refined structure (gray ribbon model) after structure determination by molecular replacement. Anomalous difference map (purple mesh; contoured at 4.0 *σ*) is also shown. (**B**) Close-up view of the HAD16 binding site in the final refined structure with 2m*F*_o_–D*F*_c_ map (cyan mesh; 1.0 *σ*). The C, N, O, I, and Br atoms of HAD16 (stick models), which is assigned as alternative conformations, are colored yellow, blue, red, purple, and brown, respectively. Alkyl chains derived from native lipid or DMPC/CHAPSO bicelles are depicted as orange stick models.

The HAD16 molecule adopting alternative conformations binds to an interface region among three symmetry-related bacteriorhodopsin molecules (Figure 8). The long alkyl tail of HAD16 interacts with the hydrophobic transmembrane surface. The choline and phosphate group face the bulk solvent. The aromatic ring in the head group ring is stabilized by π–π stacking with Y26 (Figure 8B) and hydrophobic interactions of I/Br atoms with L22, G23, V127, and L221 from a bacteriorhodopsin molecule and W80 from a neighboring molecule. Because a lipid or detergent molecule is located in the corresponding site of the native bacteriorhodopsin structure, we concluded that HAD16 binds to the same site by partially replacing the lipid or detergent. Thus, we modeled lipid/detergent-derived alkyl chains and HAD16 together using partial occupancies. We also collected a dataset from microcrystals soaked with HAD16H lacking the alkyl tail to study the importance of the tail region for bacteriorhodopsin binding (Scheme 4). The anomalous difference map did not show any significant peaks assignable to the compound (data not shown). This establishes that the alkyl chain was indispensable for HAD16 binding to bacteriorhodopsin.

## 4. Conclusions

Biomembranes are the last frontier in life science and the most challenging subject to study. Biomembranes are composed of a lipid bilayer consisting of phospholipids, glycolipids, sterols, and membrane proteins. We presented here artificial lipids and detergents containing heavy atoms, which can be used as components of model biomembranes for analyzing interactions with membrane proteins. In addition to the examples presented above, there is also a study of a detergent labeled with Hg [75]. In combination with X-ray crystallography, these artificial lipids and detergents can be used to determine novel structures of membrane proteins or to identify the orientation of lipid/detergent molecules by visualizing the position of the heavy atoms using anomalous X-ray scattering. In particular, SFX can observe damage-free structures at physiological temperatures. Future applications of these lipids/detergents for elucidating structure–function relationships of membrane proteins and biomembranes include: (i) labeling of various model lipids to distinguish between outer and inner leaflet regions that bind specifically to membrane proteins; (ii) observation of peripheral lipids surrounding membrane proteins with weak affinity at high resolution; and (iii) visualization of the dynamics of lipid localization on the surface of membrane proteins by time-resolved SFX analysis [76].

## Data Availability

Not applicable.

## Supplementary Materials

The following are available online at https://www.mdpi.com/article/10.3390/membranes11110823/s1.

## Abbreviations

A2A GPCR	G protein-coupled A2a adenosine receptor
α-GalCer	α-galactosylceramide
β-MeSe-Fuc	selenomethyl derivative of fucose
βMeSe-GlcNAc	Selenomethyl-*N*-acetylglucosamine
CHAPSO	3-[(3-cholamidopropyl)dimethylammonio]-2-hydroxypropanesulfonate
CMC	critical micelle concentration
DDM	dodecyl-β-D-maltoside
DMPC	1,2-dimyristoyl-*sn*-glycero-3-phosphocholine
DPPC	dipalmitoylphosphatidylcholine
ERATO	Exploratory Research for Advanced Technology
I3C	5-amino-2,4,6-triiodoisophthalic acid
JST	Japan Science and Technology Agency
LCLS	Linac Coherent Light Source
LCP	lipidic cubic phase
MAD	multi-wavelength anomalous diffraction
MEXT	Ministry of Education, Culture, Sports, Science and Technology
MIR	multiple heavy-atom isomorphous replacement
MIRAS	multiple heavy-atom isomorphous replacement with anomalous scattering
PAL-XFEL	Pohang Accelerator Laboratory X-ray Free Electron Laser
PDB	Protein Data Bank
PRESTO	Precursory Research for Embryonic Science and Technology
SACLA	SPring-8 Angstrom Compact Free Electron Laser
SAD	single-wavelength anomalous diffraction
SeDDM	dodecyl-β-D-selenomaltoside
SFX	serial femtosecond crystallography
SIR	single heavy-atom isomorphous replacement
SIRAS	single heavy-atom isomorphous replacement with anomalous scattering
SRX	synchrotron radiation crystallography
SwissFEL	Swiss X-ray Free Electron Laser
XFEL	X-ray free-electron laser
