# Supplementary material for: Heavy Atom Detergent/Lipid Combined X-ray Crystallography for Elucidating the Structure-Function Relationships of Membrane Proteins
Source: Membranes (Basel). 2021 Oct 27;11(11):823. doi: 10.3390/membranes11110823 (PMC8625833; doi:10.3390/membranes11110823)
Supplement: Supplementary file 1 [file membranes-11-00823-s001.zip › membranes-1398756-supplementary.pdf]

membranes

Supplementary Information

Heavy Atom Detergent/Lipid Combined X-ray Crystallography for Elucidating the Structure-function Relationships of Membrane Proteins

Shinya Hanashima [1], Takanori Nakane [2,†] and Eiichi Mizohata [3,4,*]

[1] Department of Chemistry, Graduate School of Science, Osaka University, 1-1 Machikaneyama, Toyonaka 563-0043, Japan; hanashimas13@chem.sci.osaka-u.ac.jp
[2] Department of Biological Sciences, Graduate School of Science, The University of Tokyo, Tokyo 113-0033, Japan; tnakane@mrc-lmb.cam.ac.uk
[3] Department of Applied Chemistry, Graduate School of Engineering, Osaka University, 2-1 Yamadaoka, Suita 565-0871, Japan
[4] Japan Science and Technology Agency, PRESTO, Kawaguchi 332-0012, Japan
* Correspondence: mizohata@chem.eng.osaka-u.ac.jp; Tel.: +81-6-6879-7410; Fax: +81-6-6879-7409
† Present address: MRC Laboratory of Molecular Biology, Cambridge CB2 0QH, UK.

1. DMPC/CHAPSO bicelle preparations

An appropriate amount of DMPC and heavy atom labeled compounds were dissolved in $CHCl_3$/methanol 2:1 and dried under a stream of argon gas, and the residue was dried at least for 12 h under high vacuum. An appropriate amount of CHAPSO solution (10% w/v) was added to the dried residue to fulfill the q value (DMPC/CHAPSO) = 2.8, and milliQ water was added to the solution to be 40% (w/v). Following freeze-thaw cycle was repeated until the solution became homogeneous and transparent.

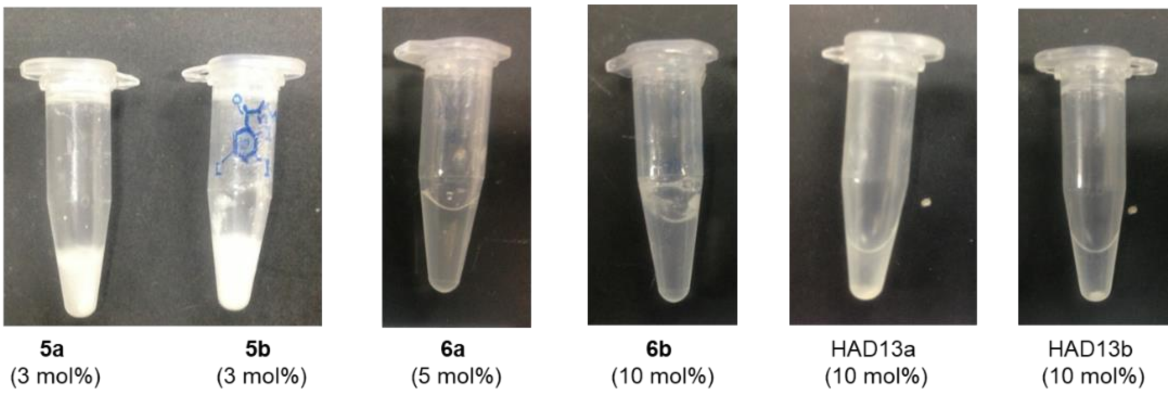

Figure S1. Miscibility of the heavy atom labeled compounds in DMPC/CHAPSO bicelles. Compound **5a** and **5b** gave white precipitates, while the others became homogeneous and transparent solutions after freeze-thaw cycles. **6a** corresponds to HAD16.

2. Bis-O-tosyl DMPC 3

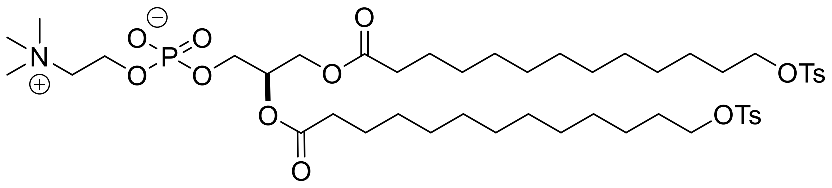

To a solution of OTs carboxylic acid (176 mg, 0.458 mmol) and *sn*-glycero-3-phosphocholine (20 mg, 0.0778 mmol) in dry CH_2Cl_2 (1 mL) added DCC (101 mg, 0.49 mmol) and 4-pyrrolidinopyridine (41 mg, 0.28 mmol) in dry CH_2Cl_2 (0.6 mL) was stirred 1 day at room temperature. The mixture was concentrated and then diluted and passed through SepPak C18 (methanol/water), and finally purified with SiO_2 chromatography (chloroform/methanol/water = 65:25:4) to give **3** in 40% yield.

white solid; Rf : 0.25 (silica gel, 65:25:4-chloroform/methanol/water); ^{1}H-NMR (500 MHz, $CDCl_3$) δ 7.82 (2H, d, *J* = 8.0 Hz), 7.49 (2H, d, *J* = 8.0 Hz), 5.28 (1H, m), 4.47(1H, dd, *J* = 12, 3.0 Hz), 4.32 (2H, d, *J* = 1.3 Hz), 4.22 (1H, dd, *J* = 12, 6.5 Hz), 4.13 (2H, t, *J* = 7.50 Hz), 4.05 (4H, t, *J* = 6.0 Hz), 3.69 (2H, t, *J* = 4.5 Hz), 3.27 (9H, s, $N(CH_3)_3$-), 2.50 (6H, s), 2.38 (2H, t, *J* = 7.5 Hz), 2.36 (2H, t, *J* = 7.5 Hz), 1.23 (40H, m).

3. Br_2-DMPC

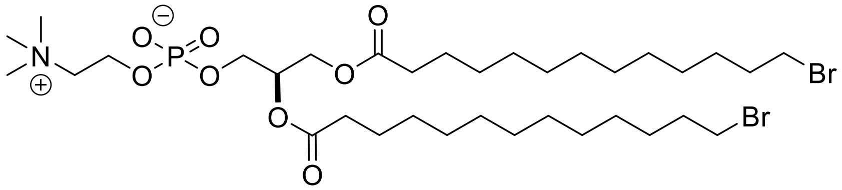

To a solution of tosylate **3** (15 mg, 0.015 mmol) in acetone (0.5 mL) added LiBr (30 mg, 0.29 mmol) was stirred 1 day at 35 °C and then filtered through Celite. The filtrate was concentrated and purified with SiO_2 chromatography (chloroform/methanol/water = 65:25:4) to give **Br_2-DMPC** in 41% yield.

white solid; Rf : 0.25 (silica gel, 65:25:4-chloroform/methanol/water); ^{1}H-NMR (500 MHz, CD_3OD) δ 5.30 (1H, m), 4.41(1H, dd, *J* = 12, 3.5 Hz), 4.25 (2H, m), 4.16 (1H, dd, *J* = 12, 6.5 Hz), 4.13 (1H, t, *J* = 7.50 H), 3.98 (1H, t, *J* = 6.0 Hz), 3.62 (2H, t, *J* = 4.5 Hz), 3.53 (2H, t, *J* = 7.0 Hz), 3.41 (2H, t, *J* = 7.0 Hz), 3.20 (9H, s, $N(CH_3)_3$-), 2.32 (2H, t, *J* = 7.0 Hz), 2.30 (2H, t, *J* = 7.5 Hz), 2.10 (4H, quin, *J* = 3.0 Hz), 1.82 (4H, quin, *J* = 7.0 Hz), 1.58 (4H, quin, *J* = 7.5 Hz), 1.43 (4H, quin, *J* = 7.0 Hz), 1.43 (4H, quin, *J* = 7.0 Hz), 1.29 (20H, m).

4. N-1-Octyl-2,3,5-triiodobenzamide

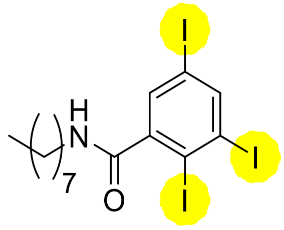

To a solution of 2,3,5-triiodobenzoic acid (0.48 g, 0.95 mmol) in dry CH_2Cl_2 (20 mL) added *n*-octylamine (0.23 mL, 1.44 mmol), EDCI·HCl (0.27 g, 1.45 mmol), and DMAP (0.17 g, 1.45 mmol) was stirred for 22 h under reflux conditions. After cooling to room temperature, the mixture was diluted with water and extracted with $CHCl_3$ (×3). The combined organic phase was washed with NH_4Cl solution and brine, dried over Na_2SO_4, filtered and concentrated. The residue was purified with SiO_2 chromatography (10:1 hexane/ethylacetate) to give N-1-octyl-2,3,5-triiodobenzamide (0.46 g, 0.76 mmol, 81%).

white powder; Rf = 0.20 (10 : 1 - Hexane : ethyl acetate) ; ^{1}H NMR (400 MHz, $CDCl_3$) δ 8.22 (1H, d, *J* = 1.6 Hz, p-Ar), 7.52 (1H, d, *J* = 1.6 Hz, o-Ar), 5.61 (1H, s, CO*NH*), 3.42 (2H, q, NH*CH₂*), 1.29 (12H, CH_2), 0.899-0.871 (3H, t, CH_3).

5. 1-O-Myristoyl-2-O-2,3,5-triiodobenzoyl-phosphatidylcholine

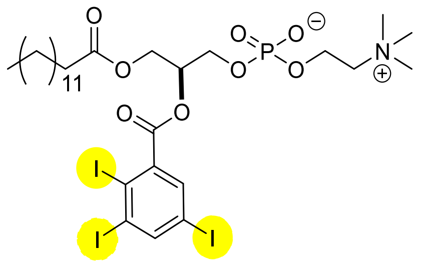

A solution of LysoPC (20 mg, 0.042 mol) and 2,3,5-triiodobenzoic acid (42.5 mg, 0.085 mmol) in dry toluene was co-evaporated and dried under reduced pressure. The residue was dissolved in dry CH_2Cl_2 (3 mL), and the solution of 2-methyl-6-nitrobenzoic anhydride (NMBA) (74 mg, 0.22 mmol) and 2-pyrrolidinopyridine (61 mg, 0.41 mmol) in dry CH_2Cl_2 (2 mL) was added. The mixture was stirred for 12 h at room temperature, and then quenched with methanol, and concentrated under the reduced pressure. The residue was purified with SiO_2 chromatography (65:25:2, chloroform/methanol/water) to give the title compound (27.1 mg, 0.028 mmol, 67%).

white powder; Rf = 0.3 (65 : 25 : 4 - chloroform : methanol : H_2O); ^{1}H NMR (400 MHz, CD_3OD) δ 8.27 (1H, t) 8.12 (2H, d, *J* = 12 Hz), 5.4 (1H, m), 4.4 (2H, m, *J* = 12, 3.2 Hz), 4.25 (2H, m), 4.15 (2H, dd, *J* = 12, 6.8 Hz), 3.98 (2H, t, *J* = 6.0 Hz), 3.62 (2H, br t, *J* = 4.8 Hz), 3.28 (9H, s), 2.32 (2H, t, *J* = 7.2 Hz), 1.58 (2H, br), 1.29-1.26 (36H, m), 0.88 (3H, t, *J* = 6.8 Hz); ESI-MS m/z = 972.01[M+Na]$^+$.

6. 1-O-myristoyl-2-O-(3-iodo-5-bromo-benzoyl)-3-sn-phosphatidylcoline (HAD16)

Before reaction, the head group of HAD16 (HAD16H), 3-iodo-5-bromo benzoic acid (80 mg, 0.25 mmol) and lysophosphatidylcholine (lysoPC a C14:0) (64 mg, 0.14 mmol) in 50 mL round bottom flask was co-evaporated with dry toluene for three times. Then, the mixture was dissolved in dry CH_2Cl_2 (3 mL), and the solution of 2-methyl-6-nitrobenzoic acid anhydride (NMBA; 110 mg, 0.32 mmol) and 4-pyrolidinopyridine (98 mg, 0.66 mmol) in CH_2Cl_2 (2 mL) was added to the reaction flask using a cannula. After stirring for 12 h at room temperature, the reaction mixture was quenched with a few drops of MeOH and solvent was removed by evaporation. The residue was purified with SiO_2 flash chromatography ($CHCl_3$:MeOH = 3:2 to $CHCl_3$:MeOH:H_2O = 65:25:2) to give HAD16 (69 mg, 0.089 mmol, 64%).

white powder; ^1H NMR (400 MHz, CD_3OD) δ 8.27 (1H, t) 8.12 (2H, d, *J* = 12 Hz), 5.4 (1H, m, H2), 4.4 (2H, m, *J* = 12, 3.2 Hz), 4.25 (2H, m), 4.15 (2H, dd, *J* = 12, 6.8 Hz), 3.98 (2H, t, 6.0 Hz), 3.62 (2H, br t, 4.8 Hz), 3.28 (9H, s), 2.32 (2H, t, *J* = 7.2 Hz), 1.58 (2H, br), 1.29-1.26 (36H, m), 0.88 (3H, t, *J* = 6.8 Hz); HR-ESI-MS calcd. C29H49BrINO8P 776.1424 $[M+H]^+$, found 776.1429.

7. Serial femtosecond crystallography (SFX) for bacteriorhodopsin with HAD16 and HAD16H

Crystallization of bacteriorhodopsin in bicelles, SFX data collection, and structure determination were performed in the same way as previously described for the HAD13a method [1]. The HAD16 bound structure was refined based on the model from the HAD13a derivative. Data statistics are shown in **Table S1**.

Table S1. Data collection and refinement statistics for bacteriorhodopsin calculated for datasets merged with Friedel's law, except for CC_{ano}.

**Dataset name**	HAD16	HAD16H
**Data collection**		
Wavelength (Å)	1.771	1.771
Space group	$C222_1$	$C222_1$
Unit cell param. *a*, *b*, *c* (Å)	46.2, 103.0, 128.7	46.2, 103.0, 128.7
No. of collected images	268,890	188,450
No. of hits	41,861	74,567
No. of indexed patterns	18,069	19,707
Indexing rate from hits (%)	43.2	26.4
Indexed pattern nos. used	18,069	19,707
No. of total reflections	2,723,979	3,061,634
No of unique reflections	13,211	13,212
Multiplicity	206.2	231.7
Resolution range (Å)	42.9–2.35	42.9–2.35

(outer shell)	(2.43–2.35)	(2.43–2.35)
Completeness (%)	100	100
R_{split} (%)	8.83 (98.9)	11.5 (51.2)
$CC_{1/2}$ (%)	99.1 (48.8)	97.1 (75.7)
CC_{ano} (%)	-3.4 (-4.5)	-1.6 (4.1)
$\langle I/\sigma(I) \rangle$	7.34 (1.08)	8.10 (2.03)
**Refinement**		
No. of reflections	12,530	
R_{work} / R_{free} (%)	15.8 / 21.0	
Mean *B*-factor (Å^2)	79.3	
No. of non-H atoms	2,067	
Rmsd from ideal bond lengths (Å) / angles (°)	0.017 / 1.955	
**PDB ID**	7VSO	

Reference

1. Nakane, T.; Hanashima, S.; Suzuki, M.; Saiki, H.; Hayashi, T.; Kakinouchi, K.; Sugiyama, S.; Kawatake, S.; Matsuoka, S.; Matsumori, N.; et al. Membrane protein structure determination by SAD, SIR, or SIRAS phasing in serial femtosecond crystallography using an iododetergent. *Proc Natl Acad Sci U S A* **2016**, *113*, 13039-13044, doi:10.1073/pnas.1602531113.
